# DEFLAZACORT VERSUS OTHER GLUCOCORTICOIDS: A COMPARISON

**DOI:** 10.4103/0019-5154.44786

**Published:** 2008

**Authors:** Surajit Nayak, Basanti Acharjya

**Affiliations:** *From the Department of Skin and VD, MKCG Medical College and Hospital, Berhampur, Orissa 760004, India*

**Keywords:** *Deflazacort*, *glucocorticoids*, *topical therapy*

## Abstract

Steroids form an important component of dermatological therapy and are used since very long time for different conditions in different forms. Though very few molecules are used since very long time, the side effect associated with this group of drugs are almost always there. Recently a new molecule deflazacort has been introduced into Indian market, is a glucocorticoid and a derivative of old molecule prednisolone. Though claimed to be having less side effect, very few studies have been done in Indian prospective. This review will highlight the very basics of this drug and its advantages and disadvantages.

## Introduction

Glucocorticoids represent the most important and frequently used class of anti-inflammatory drugs. However, unfortunately, the currently available ones act nonselectively; therefore, in the long run, they may impair many healthy anabolic processes. Various complications associated with this drug class warrant caution. Monitoring each of the drugs with separate formulation and considerable research have been focused recently on elaboration of selectively acting novel glucocorticoid drugs that possess the same efficacy in conditions for which they are used today but with reduction in one or more of the dose-limiting side effects. This article will address the therapeutic benefits of a newly introduced systemic glucocorticoid, deflazacort and its comparison with other commonly used glucocorticoids. It is a fact that very few studies and clinical trials have been performed involving this drug in dermatological context. Therefore, in this study, we will mainly discuss about this new drug in brief and will focus on whether it offers any advantages over established and commonly used corticosteroids such as prednisolone and methylprednisolone.

### Pharmacological properties

A new glucocorticoid deflazacort was introduced in 1969; it is a D-ring substituted steroid, otherwise similar to cortisol. It is a synthetic oxazoline derivative of prednisolone, having the molecular formula11beta, 21-dihydroxy-2′-methyl-5′beta-H-pregna-1, 4-dieno [17,16d] oxazole-3, 20-dione-21-acetate.

#### (A) Pharmacodynamic properties

Deflazacort is a glucocorticoid. Its anti-inflammatory and immunosuppressive effects are used in treating various diseases and are comparable to other anti-inflammatory steroids. Clinical studies have indicated that the average potency ratio of deflazacort to prednisolone is 0.69–0.89 and 6 mg of deflazacort is equivalent to 5 mg of prednisolone. However, the therapeutic dosage ratio has been reported to range from 1:1.2 to 1:1.5.^1^

#### (B) Pharmacokinetic properties

Orally administered deflazacort appears to be well absorbed and is immediately converted by plasma esterases to the pharmacologically active metabolite (D 21-OH), which achieves peak plasma concentrations in 1.5 to 2 h. It is 40% protein bound and has no affinity for corticosteroid binding globulin (transcortin) and binds to plasma protein and blood cells instead, crossing the blood-brain barrier in very low concentrations.^2^ These pharmacokinetic and biochemical properties of deflazacort may prevent it from reaching the hypothalamic or pituitary circulation during the first years; however, we can speculate that after a long-term treatment, deflazacort levels could increase in the central nervous system and finally produce effects similar to other glucocorticosteroids.^3^ Its plasma elimination half-life is 1.1 to 1.9 h. Elimination takes place primarily through the kidneys; 70% of the administered dose is excreted in the urine, and the remaining 30% is eliminated through the feces. Metabolism of D 21-OH is extensive; only 18% of urinary excretion represents D 21-OH. The metabolite of D 21-OH, deflazacort 6-beta-OH, represents one-third of the urinary elimination. Due to the short pharmacokinetic half-life of its active metabolite, pharmacodynamic effects of deflazacort are of shorter duration than those of methylprednisolone and prednisolone.

#### (C) Preclinical safety data

Safety studies have been carried out in the rat, dog, mouse and monkey. The findings are consistent with other glucocorticoids at comparable doses. Teratogenic effects demonstrated in rodents and rabbits are typical of those caused by other glucocorticoids. Deflazacort was not found to be carcinogenic in the mouse, but studies in the rat produced carcinogenic findings consistent with the findings obtained using other glucocorticoids.

#### Indications and clinical uses

It is indicated for various different conditions as indicated in other glucocorticoids and has demonstrated equivalent efficacy to prednisolone and other oral steroids in rheumatoid arthritis, nephritic syndrome, SLE, transplantation, polymyalgia rheumatica, sarcoidosis and juvenile chronic arthritis. Although very few clinical trials have been performed under various dermatological conditions, it is shown to be effective in various steroid responsive cases.

#### Warning and precautions

Being a derivative of prednisolone, same precaution should be exercised as for other glucocorticoids. As it is metabolized in liver, it is recommended to increase the maintenance dose of deflazacort if drugs, which are liver enzyme inducers, are co-administered.

#### Pregnancy and lactation

The ability of corticosteroids to cross the placenta varies between individual drugs; however, deflazacort does cross the placenta. As with all drugs, corticosteroids should only be prescribed when the benefits to the mother and child outweigh the risks. When corticosteroids are essential, however, patients with normal pregnancies may be treated as if they were in the nongravid state. Corticosteroids are excreted in breast milk, although no data are available for deflazacort. Doses of up to 50 mg daily of deflazacort are unlikely to cause systemic effects in the infant. Infants of mothers taking higher doses than this may have a degree of adrenal suppression, but the benefits of breast-feeding are likely to outweigh any theoretical risk.

#### Doses and administration

Deflazacort is a glucocorticoid derived from prednisolone and 6 mg of deflazacort it has approximately the same anti-inflammatory potency as 5 mg prednisolone or prednisone. Doses vary widely in different diseases and different patients. In more serious and life-threatening conditions, high doses of deflazacort may need to be given. When deflazacort is used for long term in relatively benign chronic diseases, the maintenance dose should be kept as low as possible. Dosage may need to be increased during periods of stress or during exacerbation of illness. The dosage should be individually titrated according to diagnosis, severity of disease and patient response and tolerance. The lowest dose that will produce an acceptable response should be used. In patients with hepatic impairment; blood levels of deflazacort may be increased. Therefore, the dose of deflazacort should be carefully monitored and adjusted to the minimum effective dose. In renal impairment, no special precautions other than those usually adopted in patients receiving glucocorticoid therapy are necessary. In elderly patients, no special precautions other than those usually adopted in patients receiving glucocorticoid therapy are necessary. There has been limited exposure of children to deflazacort in clinical trials. In children, the indications for glucocorticoids are the same as for adults, but it is important that the lowest effective dosage is used. Alternate day administration may be appropriate. Doses of deflazacort usually lie in the range 0.25–1.5 mg/kg/day.

For acute disorders up to 120 mg/day, deflazacort might be administered initially. The maintenance dose is usually within range 3–18 mg/day. The smallest effective dose should be used and increased if necessary.

#### Deflazacort withdrawal

In patients who have received more than physiological doses of systemic corticosteroids (approximately 9 mg per day or equivalent) for greater than 3 weeks, withdrawal should not be abrupt. How dose reduction should be carried out largely depends on whether the disease is likely to relapse as the dose of systemic corticosteroids is reduced. Clinical assessment of disease activity might be needed during withdrawal. If the disease is unlikely to relapse on withdrawal of systemic corticosteroids, but there is uncertainty regarding HPA suppression, the dose of systemic corticosteroids can be reduced rapidly to physiological doses. Once a daily dose equivalent to 9 mg deflazacort is reached, dose reduction should be slower to allow the HPA-axis to recover.

Abrupt withdrawal of systemic corticosteroid treatment that is continued up to 3 weeks is appropriate if it is considered that the disease is unlikely to relapse. Abrupt withdrawal of doses up to 48 mg daily of deflazacort or equivalent for 3 weeks is unlikely to lead to clinically relevant HPA-axis suppression in the majority of patients. In the following patient groups, gradual withdrawal of systemic corticosteroid therapy should be *considered* even after courses lasting for 3 weeks or less:

- Patients who have had repeated courses of systemic corticosteroids, particularly if taken for greater than 3 weeks.
- When a short course has been prescribed within one year of cessation of long-term therapy (months or years).
- Patients who may have reasons for adrenocortical insufficiency other than exogenous corticosteroid therapy.
- Patients receiving doses of systemic corticosteroid greater than 48 mg daily of deflazacort (or equivalent).
- Patients repeatedly taking doses in the evening.

#### Overdosage

It is unlikely that treatment is needed in cases of acute overdosage.The LD50 for the oral dose is greater than 4000 mg/kg in laboratory animals.

#### Comparison with other glucocorticoids

An important issue to be addressed when comparing two glucocorticosteroids is their respective potency. Fewer side effects may be related to less primary activity. The bioequivalence of deflazacort and prednisolone has been investigated in various situations. In normal subjects,^4^ 15 mg deflazacort inhibits T cell reactivity to the same extent as 12.5 mg prednisolone, but for a longer period of time (based on phytohemagglutinin-induced T cell proliferation *in vitro*). Based on the results of seven trials of various design, including double blind cross-over studies, paired patient studies, and between-patient studies involving 160 patients, the potency ratio of deflazacort *vs.* prednisolone was estimated to be 1.28 by Avioli.^5^ In fact, the equivalence ratio of deflazacort/prednisolone may depend on the disease [i.e., 1.2:1 in rheumatoid arthritis,^6^ juvenile chronic arthritis^7^ and nephrotic syndrome,^8^ and 1.4:1 in asthma^9^ and polymyalgia rheumatica.^10^ In a study comparing deflazacort *vs.* methylprednisolone using a 1.5:1 ratio (which is equivalent to a 1.2:1 deflazacort/prednisolone ratio), Elli *et al*.^11^ concluded that there was a more potent immunosuppressive activity of deflazacort with a lower ratio of CD4^+^/CD8^+^ lymphocytes. It has been suggested that deflazacort depresses the osteoblast less than prednisolone, leading to a smaller decrease in serum osteocalcin levels with this drug.^12^ A bone-sparing effect of deflazacort at the level of lumbar spine has been described in various clinical situations. In a 1-year double blind prospective study of patients with the nephrotic syndrome, bone loss induced by prednisolone at the lumbar spine was 1.9-fold higher (12.5%/year) than that induced by deflazacort (6.8%).^13^ Other researchers have claimed that some of the bone-sparing effect of deflazacort compared to that of prednisolone could be explained by a less impaired intestinal calcium absorption by the former.^14^,^15^ In general, deflazacort appears to have less effect than prednisone on parameters that may be associated with the development of corticosteroid-induced osteoporosis. Further, the drug appears have less negative impact on growth rate in children with diseases requiring corticosteroid therapy. Thus, deflazacort may be associated with less serious metabolic sequelae than prednisone, but further well designed long-term trials are required to confirm this. Histomorphometry and densitometry techniques have shown that when used at doses with approximately equivalent anti-inflammatory efficacy, it appears to have fewer detrimental effects on bone mass than prednisone.

In children, however, even though the available efficacy data are minimal, deflazacort should be considered as an initial option in those requiring corticosteroid therapies since the adverse effects caused by this drug class are particularly debilitating in this patient group. Gastrointestinal symptoms are the most frequently reported adverse events in deflazacort recipients; other adverse events associated with the drug include metabolic and nutritional disorders, central and peripheral nervous system disturbances and psychiatric disorders. Past reports have indicated that deflazacort is less diabetogenic than prednisone in healthy subjects.^16^,^17^ However, in a recent study, it has also been reported to be show similar effect on glucose tolerance.^18^ Different studies also shows that a long-term treatment with deflazacort has a smaller effect on glucose metabolism than betamethasone.^19^ Another study reinforces the finding by obtaining a result that indicates that betamethasone-induced greater glucose intolerance and insulin resistance than deflazacort.^20^ These results indicate that deflazacort when employed in an anti-inflammatory dose equivalent to prednisone should prove advantageous in insulin-treated diabetics who require steroid treatment. Various studies have shown that using deflazacort instead of prednisolone in kidney transplant patients helps to prevent fat accumulation and worsening of lipid profile;^21^ This is similar to the result of another study involving methylprednisolone, which has shown that the use of deflazacort as a maintenance therapy prevents fat accumulation and lead to an improvement in the lipoproteins.^22^ The overall incidence of adverse events in deflazacort recipients (16.5%) is lower than that recorded in patients treated with prednisone (20.5%) or methylprednisolone (32.7%) and similar to that in betamethasone recipients (15.3%).^23^

## Summary

Following clinical trials over more than 20 years, it has been approved in many countries (Italy, France, UK, Germany, and Spain) for use in inflammatory diseases. With regard to its effect, it has been claimed to have, at doses with equivalent anti-inflammatory efficacy to prednisolone, less severe adverse effects on bone, carbohydrate metabolism and linear growth and with less GI side effects. However, these claims have been questioned on the basis of some doubts as to the dose equivalence of deflazacort and the glucocorticoid of reference, prednisolone. Most of the data on the bone sparing effect of the drugs are obtained from trials that are relatively small or of short duration. Therefore, well-designed clinical trials are needed, especially to clarify the appropriate ratio of doses for bioequivalence with prednisone. Unfortunately, very few studies have been performed by various authors on a small number of patients with different results; further, open study design makes it difficult to interpret the clinical relevance of those studies. Most important studies of deflazacort in various dermatological conditions is almost negligible, and no opinion or comment can be expressed regarding its advantages over other glucoorticoids in treating various diseases at this juncture. As learnt from the available literature, it can be stated that deflazacort has no advantages over prednisolone in short-term use; further, on the basis of current evidence provided in present studies, it is not possible to confirm whether the trend shown in trials of lower risk in long-term side effects will be borne out in larger trials or in clinical practice. However, we can state that it is worth considering those on long-term steroids, who are at higher risk of metabolic side effects, and in children as an initial option in those requiring corticosteroid therapies since the adverse effects caused by this drug class are particularly debilitating in this patient group. Such patients will be usually under specialist care, and this drug should be reserved for specialists only. Long-term study and trials in dermatological conditions are required before we can provide any concrete opinion regarding its utility for our domain.
